# Response to the Letter to the Editor Regarding ‘Lived Experiences of Returning to Participation After Mild Stroke: A Phenomenological Study in Spain’

**DOI:** 10.1111/hex.70970

**Published:** 2026-11-11

**Authors:** C. de Diego‐Alonso, A. Buesa Estéllez, J. Güeita‐Rodriguez, P. Bellosta‐López, P. Roldán‐Pérez

**Affiliations:** ^1^ Universidad San Jorge, MOTUS Research Group Villanueva de Gállego Zaragoza Spain; ^2^ Department of Physiotherapy, Occupational Therapy, Rehabilitation, and Physical Medicine, Research Group of Humanities and Qualitative Research in Health Science (Hum&QRinHS) Universidad Rey Juan Carlos Madrid Spain

**Keywords:** patient participation, return period, stroke

Dear Editor,

We sincerely thank the authors for their careful reading and thoughtful comments and welcome the opportunity to clarify the following aspects.

First, regarding the heterogeneity in time since stroke, we acknowledge that our participants represented a broad range of post‐stroke experiences. Our goal was not to compare experiences according to time since stroke or to examine recovery trajectories; rather, we sought to describe the return to participation beyond the first 6 months, when people with mild sequelae are presumed clinically stable, and their long‐term needs are largely overlooked in clinical follow‐up and research [1]. In descriptive phenomenology, participants are selected because they have lived the phenomenon, and variation in their circumstances helps to identify its invariant structure rather than introducing bias.

Consequently, time since stroke was treated descriptively rather than analytically, and no distinct thematic structures emerged according to it. Participants did not frame their experiences in terms of clinical time; instead, they emphasised self‐efficacy, social support, environmental adaptations and role reconstruction. Similar accounts of ongoing progress emerged at markedly different time points: ‘I realize how much I've progressed, and I don't want to stop’ (P15, 10 months post‐stroke); ‘the recovery is not complete ‐ it is still going on’ (P18, 6 years post‐stroke) (Supporting Information IV [1]).

This is consistent with evidence that time since stroke plays a secondary role compared with the multifactorial influences shaping participation (see Discussion [1]).

Second, participants with caregiver support maintained high levels of functional independence, as reflected by their Barthel Index scores (range 70–100, comparable to participants without support; Supporting Information II [1]). Given the selection criteria, the sample did not require a caregiver in clinical terms; this variable rather reflected a subjectively perceived need for support. This highlights the distinction between autonomy, the capacity to decide and independence, the capacity to perform without assistance. Participants described support persons who preserved their autonomy: ‘they give me some room to push myself as much as I can’ (P07, with caregiver support; Supporting Information IV [1]). Living arrangements reflect household composition rather than dependence: three of the six participants living alone also reported caregiver support.

Third, regarding caregivers' perspectives, our study was specifically designed as a phenomenological exploration of the lived experiences of stroke survivors themselves. Including caregivers would have addressed a different research question. Moreover, proxy accounts could have overshadowed the first‐person voice that phenomenology seeks to capture. Notably, participants rarely referred to a ‘caregiver’, describing instead a need for close support from family and friends (see Discussion [1]). Future focus groups or dyadic interviews with stroke survivors and support persons are needed.

Fourth, regarding the term ‘mild stroke’, we acknowledge that this term may generate confusion, as it usually refers to acute stroke severity (e.g., National Institutes of Health Stroke Scale). In our study of chronic stroke survivors, ‘mild’ was intended to describe the severity of residual sequelae. This was operationally defined by the inclusion criteria of the Part&Sed‐Stroke project [2]: independent ambulation (Functional Ambulation Categories ≥ 3) and absence of cognitive and communication impairment. According to Barthel Index cut‐off points [3], the sample would be more precisely described as chronic stroke survivors with slight‐to‐moderate dependency: 14 participants scored 100, 11 scored 91–99 and 10 scored 61–90 (Supporting Information II [1]). Far from being contradictory, the coexistence of high functional scores with perceived support needs and participation restrictions is precisely the phenomenon our study sought to explore. The Barthel Index captures basic activities of daily living, but not instrumental, social or community participation. We agree that the term ‘mild stroke’ should be reported and qualified more clearly in future studies.

Fifth, concerning the sex distribution of the sample, we acknowledge the predominance of male participants (23 men, 12 women). This proportion (34% women) mirrors that of the parent Part&Sed‐Stroke cohort (35% women) [4], indicating that it reflects the recruited population rather than a selection bias. It is also consistent with epidemiological evidence: women's relative protection against stroke before menopause results in a male predominance at younger ages [5], and most participants (31 of 35) had their stroke before the age of 65 (Supporting Information II [1]). Moreover, women tend to experience greater post‐stroke dependency, which may further reduce their representation among survivors with mild sequelae. Nevertheless, we agree that the statement in our Discussion was insufficiently supported, and that this imbalance should have been explicitly acknowledged as a limitation. Future studies should purposively seek profiles according to age, sex and time since stroke, and specifically explore potential sex‐related differences in experiences of participation after stroke.

Finally, regarding sampling, participants were recruited consecutively from eligible individuals within the larger Part&Sed‐Stroke project [2], conducted across 20 centres in different Spanish regions. Sampling was purposive in that phenomenology requires participants with firsthand experience of the firsthand experience of the phenomenon (refs. 27 and 31 in [1]) Thus, both approaches were complementary rather than alternative: the eligibility criteria defined the experience sought, and consecutive recruitment determined how eligible individuals were invited. We recognise that the manuscript did not sufficiently distinguish between both levels. Sample adequacy was determined when interviews became repetitive, and no substantially new relevant information emerged, a criterion formerly termed data saturation (ref. 31 in [1]). The resulting 35 interviews (1194 minutes; 416 units of meaning) exceed the sample sizes usually recommended for phenomenological studies.

Sincerely,

The Authors.

## Author Contributions

**C. de Diego‐Alonso:** conceptualization, writing – original draft, methodology, supervision, writing – review and editing. **A. Buesa Estéllez:** methodology, writing – review and editing, formal analysis, supervision. **J. Güeita‐Rodriguez:** writing – review and editing; validation. **P. Bellosta‐López:** conceptualization, methodology, supervision, formal analysis, writing – review and editing, writing – original draft. **P. Roldán‐Pérez:** writing – original draft, writing – review and editing, methodology, resources.

## Funding

The authors have nothing to report.

## Conflicts of Interest

The authors declare no conflicts of interest.

## Data Availability

Data sharing not applicable to this article as no datasets were generated or analysed during the current study.
